# Surgery for Non-Small Cell Lung Cancer in the Personalized Therapy Era

**DOI:** 10.3390/curroncol30080563

**Published:** 2023-08-21

**Authors:** Marco Chiappetta, Carolina Sassorossi, Giacomo Cusumano

**Affiliations:** 1 Thoracic Surgery, Fondazione Policlinico Universitario A. Gemelli IRCCS, 00168 Rome, Italy; sassorossi.caro@gmail.com; 2 Department of Translational Medicine and Surgery, Università Cattolica del Sacro Cuore, 00168 Rome, Italy; 3 Thoracic Surgery Unit, Policlinico—San Marco Hospital, University of Catania, 95124 Catania, Italy; giacomo.cusumano@unict.it

Lung cancer remains one of the tumours with the highest incidence and the poorest prognosis, with an estimated incidence of more than 220,000 cases with 135,000 cancer-related deaths annually in the United States [1,2].

As such, the diagnostic and therapeutic approach should be considered multidisciplinary, involving not only different medical specialties but also incorporating health care policy to reduce cancer development risks and enhance early-stage detection.

Lung cancer screening programs are effective in reducing cancer-related mortality in specific high-risk patient groups. Consequently, it is now recommended to develop specific screening programs within institutions that also ensure the provision of quality treatment for any discovered tumours and nodules [3,4,5].

Over the past 10–15 years, the surgical approach to early-stage non-small cell lung cancer (NSCLC), particularly in stage I tumours, has undergone significant changes, and technical aspects continue to evolve. 

Although it is now established that patients should be treated with minimally invasive techniques whenever possible [6,7], the extent of parenchymal resection remains a subject of ongoing discussion. In detail, two recent trials, the JCOG0802/WJOG4607L and the CALGB140503 trial [8,9], reported the non-inferiority or the superiority of segmentectomy compared to lobectomy in terms of the overall survival for peripheral NSCLC tumours measuring < 2 cm.

Notably, the International Association for the Study of Lung Cancer (IASLC) has addressed the need for skills and instrumentation that enable the execution of anatomical segmentectomy in cases where pure ground glass opacity is detected during screening [4].

However, some issues need to be clarified, such as the significantly higher local recurrence rate in the segmentectomy group compared to the lobectomy group in the JCOG0802/WJOG4607L trial [9]. Additionally, important information regarding the type of segmentectomy performed, whether single or multiple, was not specified. Furthermore, the rationale for choosing segmentectomy over lobectomy lies in the potential to preserve parenchyma, especially in functionally compromised patients. However, the trial’s reported FEV1 difference between the two groups was only marginal, approximately 2.7% at 6 months and 3.5% at 12 months, in favour of the segmentectomy group. This finding raises doubts about the clinical significance of such a small difference and questions the true advantage of segmentectomy over lobectomy. 

In light of these uncertainties, it is essential to critically assess the benefits and drawbacks of both approaches to ensure that the chosen role of surgery in stage treatment aligns with the best possible outcome for patients. Further research and analysis may be required to fully understand the implications of these findings and to guide treatment decisions effectively.

Patient evaluation remains a fundamental part of the treatment strategy, with the possibility of determining the appropriate therapy in early-stage tumours. Indeed, despite the surgical resection being considered the treatment of choice, other treatments, such as Stereotactic Ablative Radiotherapy (SABR), may be considered if surgery is refused or if patient comorbidities make the surgical approach extremely risky [10,11]. For this reason, respiratory and cardiac evaluations need to be modulated on patients’ characteristics and treatment options, identifying the most appropriate treatment with the highest potential advantage in terms of survival while minimizing the risk of complications [6]. 

This complex approach applies to patients with tumours potentially curable by surgery, enabling an improvement in overall survival. However, in the future, more information on histology, performance status, and stage will indicate the most suitable treatment strategy.

Indeed, in the next future, Artificial Intelligence could integrate and manage complex models based on multi-omics domains (radiological, biological, etc.), providing personalized therapies also suggesting adjuvant or biologic therapy also for early stages [12,13,14]. These advancements may potentially revolutionize the field and further enhance treatment outcomes.

On the other hand, the role of surgery in stage III patients remains an intriguing and debated issue, even though it is now clear that surgery should be considered only in a multi-disciplinary approach setting, including oncologists or radiotherapists. In particular, the treatment strategy in patients with discrete mediastinal nodal involvement remains controversial. 

In this clinical scenario, various possible strategies are proven effective and significant, such as neo-adjuvant therapy plus surgery, upfront surgery followed by adjuvant therapy, or definitive chemo-radiotherapy [7,15,16].

However, one of the most critical considerations is assessing limited N2 disease, preferably involving a single N2 station, which may guide the multidisciplinary team in considering surgery as part of the treatment. 

From this perspective, it is mandatory to have information not only on the histological classification but also on the mutational status or the PDL1 expression. Recent studies have shown the possibility of using targeted therapy as neoadjuvant therapy, but the schedule, dose, and timing of surgery are yet to be defined. For instance, Lococo et al. reported a multicentric experience in salvage surgery in ALK-rearranged NSCLC patients treated with Alectinib, achieving a complete and major pathological response in the 50% and 90% of cases, respectively [17].

Based on these preliminary results, neo-adjuvant protocols are rapidly changing, disrupting the historical platinum-based chemotherapy and introducing new possible adoption of immunotherapy, biological agents and other drugs based on cancer biology and mutational status. However, most results are derived from limited case series, while prospective trials are still ongoing, waiting for definitive results [18,19]. Nevertheless, preliminary results are encouraging.

This new perspective will open a novel scenario that demands more comprehensive information from diagnostic biopsies, including the possibility of performing next-generation sequencing to identify potential mutation targets for specific drugs. Consequently, in the future, we will require more tissue during diagnostic exams, or technology will be able to analyze imaging giving potential information on this status. In this setting, surgery is a fundamental part of this multidisciplinary management, offering the opportunity to definitely eradicate the disease and providing valuable information on the final pathological stage. Indeed, a complete response may be used as a surrogate marker for survival outcome, and information about the final nodal status, such as persistent N2 disease or lymph node ratio, may better define the prognosis and lead to adjuvant treatments [20]. 

On the flip side, there are limited insights into the intraoperative status after neoadjuvant therapy with new agents, with the risk of finding sticky tissue among nodal stations, which may render lymphadenectomy and pathological stage assessment risky or even impossible.

This Special Issue aims to explore the current status of surgery for non-small cell lung cancer, providing information about the treatment techniques and outcomes in patients with stage I–III while also collecting data regarding patient selection and technological innovations. 
